# Secreted Frizzled Related Proteins in Cardiovascular and Metabolic Diseases

**DOI:** 10.3389/fendo.2021.712217

**Published:** 2021-08-20

**Authors:** Hua Guan, Jin Zhang, Jing Luan, Hao Xu, Zhenghao Huang, Qi Yu, Xingchun Gou, Lixian Xu

**Affiliations:** ^1^State Key Laboratory of Military Stomatology & National Clinical Research Center for Oral Diseases & Shaanxi Engineering Research Center for Dental Materials and Advanced Manufacture, Department of Anethesiology, School of Stomatology, Fourth Military Medical University, Xi’an, China; ^2^Shaanxi Key Laboratory of Ischemic Cardiovascular Disease, Institute of Basic and Translational Medicine, Xi’an Medical University, Xi’an, China; ^3^Department of Preventive Medicine, School of Stomatology, Fourth Military Medical University, Xi’an, China; ^4^Shaanxi Key Laboratory of Brain Disorders & Institute of Basic and Translational Medicine, Xi’an Medical University, Xi’an, China; ^5^Institution of Basic Medical Science, Xi’an Medical University, Xi’an, China

**Keywords:** SFRPs, adipocyte, obesity, metabolism disease, cardiovascular disease

## Abstract

Abnormal gene expression and secreted protein levels are accompanied by extensive pathological changes. Secreted frizzled related protein (SFRP) family members are antagonistic inhibitors of the Wnt signaling pathway, and they were recently found to be involved in the pathogenesis of a variety of metabolic diseases, which has led to extensive interest in SFRPs. Previous reports highlighted the importance of SFRPs in lipid metabolism, obesity, type 2 diabetes mellitus and cardiovascular diseases. In this review, we provide a detailed introduction of SFRPs, including their structural characteristics, receptors, inhibitors, signaling pathways and metabolic disease impacts. In addition to summarizing the pathologies and potential molecular mechanisms associated with SFRPs, this review further suggests the potential future use of SFRPs as disease biomarkers therapeutic targets.

**Graphical Abstract:**
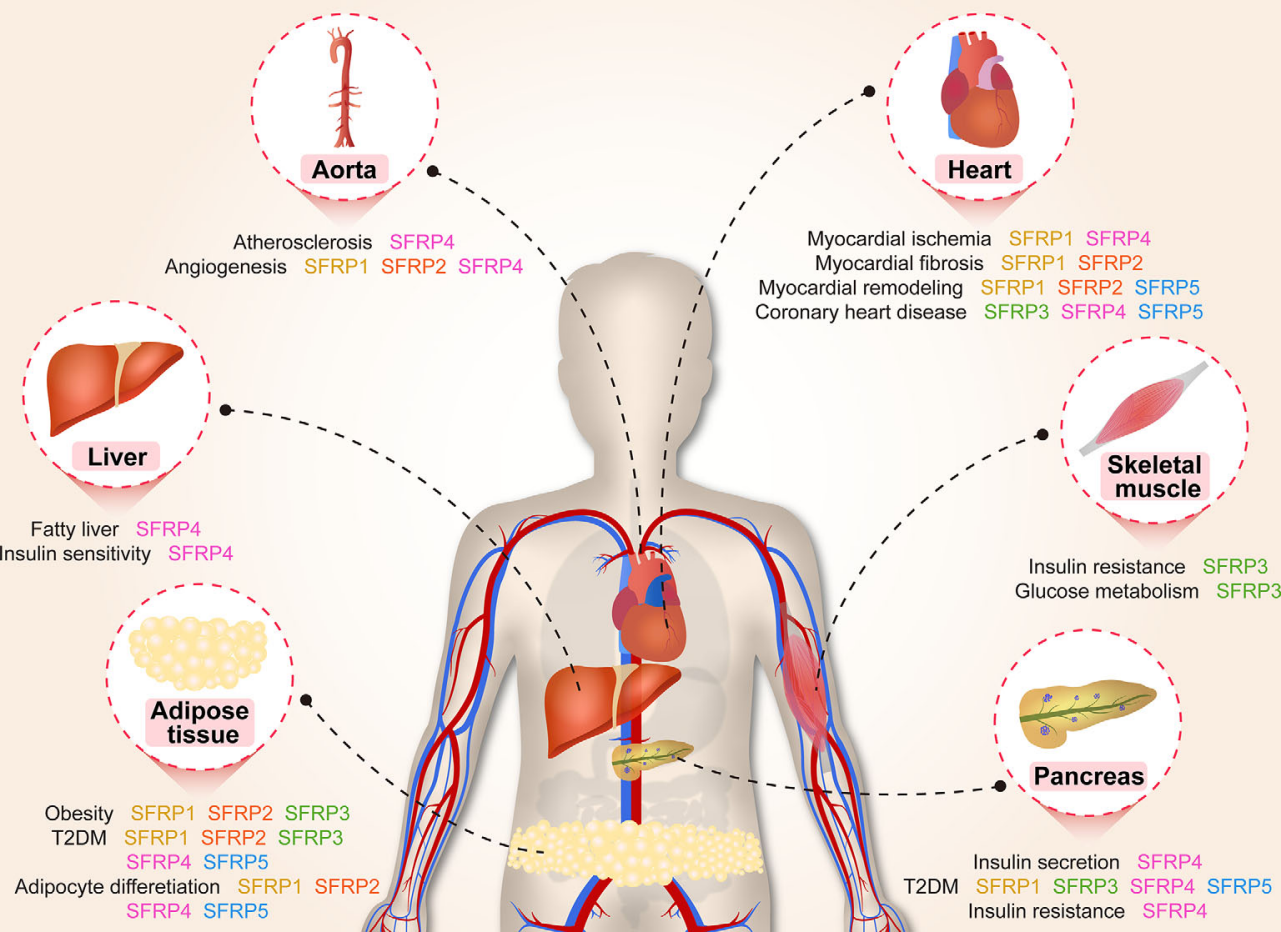


## Introduction

Metabolic syndrome (MetS) is a global epidemic that causes heavy social and economic burdens (1), and it consists of a group of cardiovascular risk factors, including dyslipidemia, glucose metabolism disorders, visceral obesity and hypertension (2). The risk of cardiovascular disease is directly proportional to the components of MetS, including the occurrence of coronary heart disease (CHD), atherosclerotic cardiovascular disease and type 2 diabetes mellitus (T2DM) (2). Central obesity, elevated blood pressure, dyslipidemia, elevated fasting blood glucose and insulin resistance (IR) are the core manifestations of the syndrome (3).

In 1973 while screening mutations for visual phenotypes, Sharma reported that the wingless gene affects the development pattern of *Drosophila* (4). Subsequently, a series of experimental studies revealed that Wnt family signaling proteins are critical for morphological development of individual embryos, including central nervous system, control of asymmetric cell division and tissue polarity (5). Wnt signal transduction is regulated by a variety of effectors, including agonists and antagonists, which fine tunes Wnt signaling to regulate intracellular signal transduction and/or extracellular ligand receptor interaction (6, 7).

With the gradual discovery of Wnt proteins, a secreted protein that binds to Wnt protein and inhibits its function was identified and named secreted frizzled related protein (SFRP) due to its high similarity with the extracellular receptor domain of frizzled (Fzd) (4). To date, five SFRP members have been identified and named SFRP1-5, all of which bind specifically with Wnt ligands (8). Multiples studies identified SFRPs as important regulators of MetS, including adipocyte differentiation, lipid metabolism, diabetes and cardiovascular diseases (9–11). In this review, we focus on the new findings implicating SFRPs in the development of metabolic diseases. We describe the structure, function, signal transduction pathways of SFRPs and their new identified functions in lipid metabolism and cardiovascular disease.

## The Characteristic and Expression Pattern of SFRPs

### The Structural Characteristic of SFRPs

**Figure 1:**
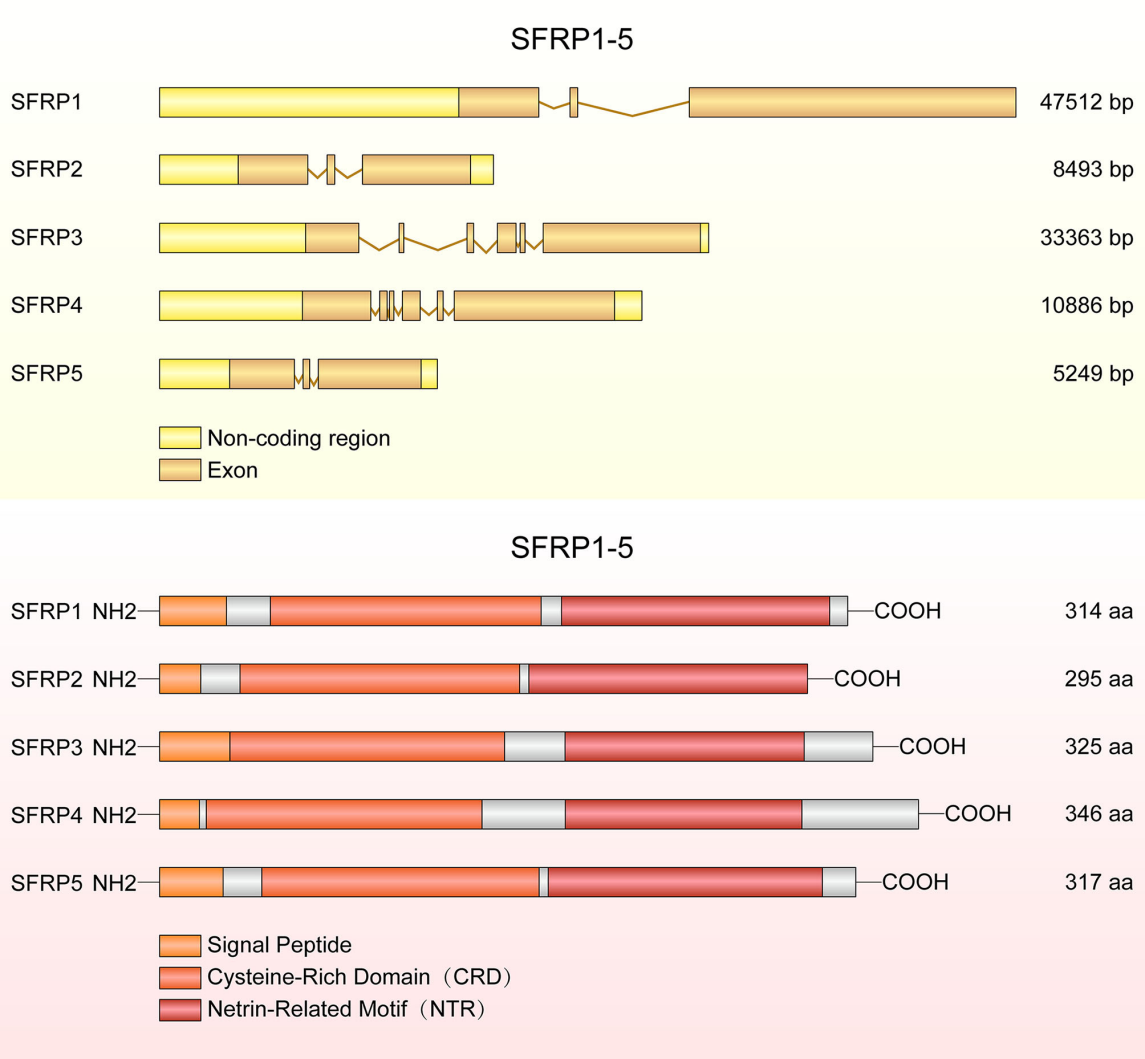
The structure of *SFRP* genes and proteins. The diagram shows *SFRP1*, *SFRP2* and *SFRP5* each containing three exons, but *SFRP3* and *SFRP4* containing six exons. SFRPs have common structural features with a signal peptide comprising 20-30 amino acids, a cysteine rich domain (CRD) and a netrin like domain (NTR).

In 1997, Ratter et al. discovered a family of mammalian genes which encodes secretion related proteins (4). These proteins are homologous to the cysteine rich ligand binding domain found in the Fzd transmembrane receptor family (4). SFRPs contain three structural units: an amino terminal signal peptide, a coiled cysteine rich domain (CRD) and a carboxyl terminus netrin-like domain (NTR) (12). The CRD spans about 120 amino acids and contains 10 conserved cysteine residues, and it presents approximately 30-50% sequence similarity with the CRD of the Fzd receptor (13). The NTR consists of six cysteine residues, several conserved hydrophobic residues and secondary structures (14). The conserved domains and structural characteristic details of SFRP1-5 are shown in **Figure 1**.

The CRD region of SFRP1 contains 10 conserved cysteine residues and forms 5 disulfide bonds. Deletion of 48 amino acids (d115-163) in CRD region decreased its activity by 75% and deletion of 92 amino acids (d71-163) resulted in complete loss of SFRP1 function, suggesting the conserved domain is essential for SFRP1 biological function (13). In addition, the motif L/V-VDGRW-L/V and DGR constitute the core of the SFRP1 binding motif, indicating that proteins containing DGR motifs may be new binding partners of SFRP1 (15). Moreover, the pocket finder was used to determine the active sites of SFRP4, including site 1 containing the amino acids, which are essential for suppressing the secretion of insulin (16). Four splice variants of SFRP4 were identified and the transcripts of all splice variants are similar at the 5 ′ end but different at the 3′ end. Markedly, the tissue distribution of the splice variants was different, suggesting that these splice variants of SFRP4 have specific overlapping functions in different tissues (17). Previous studies reported a total of 15 SFRP5 mutants, including 10 bearing non-synonymous amino acid mutations with variation frequencies closely related to the obesity phenotype (18).

In silico analyses of amino acid sequences reveal three subgroups of SFRPs: SFRP1/SFRP5, SFRP2 and SFRP3/SFRP4 (12, 19). However, sequence homology and phylogenetic tree analyses showed that SFRP1, 2 and 5 form a subgroup apart from SFRP3 and 4 (13). This cluster analysis also reflects differential gene expression of SFRPs in tissues. SFRP1, SFRP2 and SFRP5 are in different horizontal groups of the same chromosome, while SFRP4 and SFRP3 do not belong to this group. A third subgroup of SFRPs was found in *Xenopus laevis*, chicken and zebrafish but not in mammals. The members of this subgroup include sizzled, sizzled2 and crescent, which share the same sequence similarity with the SFRP1, SFRP2 and SFRP5 subgroups (20). Excitingly, tyrosine 73 of SFRP1 plays a key role in antagonizing Wnt and is conserved in the closely related SFRP1, SFRP2 and SFRP5 but replaced by tryptophan in SFRP3 and SFRP4 (13). Previous studies demonstrated that heparin inhibits tyrosine sulfation of SFRP1, and a sequence analysis demonstrates that tyrosine sulfation is highly conserved in SFRP1 and SFRP5, but not in SFRP2, SFRP3 and SFRP4 (21). Hence, the tyrosine 73 is the key tyrosine residue that binds the Fzd receptor and antagonizes the Wnt signaling pathway, whereas SFRP1 accumulates in cells and is more stability when it inhibits posttranslational modifications by inhibiting the tyrosine sulfation. Protein structure determines biological function during cell metabolism and development, proteins with similar structure are more similar in function, the following review will elaborate in detail.

### Expression Pattern of SFRPs

The differential tissue-specific expression of SFRPs in humans was verified by RNA sequencing (22). SFRP1 is ubiquitously expressed in almost all tissues, with high levels in the endometrium, fat tissue, gallbladder, heart, kidney, prostate, testis, urinary bladder and moderate levels in the ovary, may contribute to the development of reproductive system development and germ cell maturation (23, 24). SFRP2 is highly expressed in the urinary bladder, gallbladder, fat tissue and esophagus; moderately expressed in skin, small intestine, colon and appendix; not detected in heart, kidney, liver and spleen, suggesting that SFRP2 participates in the regulation of biological processes that include cell proliferation, differentiation, apoptosis, and cell localization (24). SFRP3 is expressed in almost all tissues with high levels in spleen, gall bladder, and low levels in fat tissue, bone marrow, liver and pancreas, indicating SFRP3 may be related to human immunity and the formation of immune cells (24). SFRP4 is highly expressed in the female reproductive system, such as the endometrium and ovary, moderately expressed in fat tissue, heart and urinary bladder, indicating that SFRP4 is related to female reproduction and development (24, 25). SFRP5 is highly expressed in duodenum, pancreas, small intestine, moderately expressed in the lungs, liver, gall bladder, adrenal, prostate and stomach, and dysregulation of SFRP5 in pancreas contributes to insulin secretion or glucose metabolism disease (24).

SFRPs gene expression profiles were examined in the pancreas of mice at E12-E17, P0 and P7 (26). The level of SFRP1 was high at E12-E14 and then decreased. The expression of SFRP2 started to show at E12, peaked at E14 and then decreased. SFRP3 was expressed at low levels during development and remained unchanged. SFRP4 was expressed at very low levels during development but peaked at P7, suggesting that the expression pattern of SFRPs in the pancreas is varies during the mouse embryonic development (26).

### Receptor and Inhibitors of SFRPs

SFRPs promote or inhibit Wnt/β-catenin signaling pathway, depending on the cell environment, concentration and Fzd receptor expression pattern (27). For example, SFRP1 and SFRP2 inhibit the activity of Wnt3a and regulate the development of the dorsal neural tube (28). Whereas SFRP2 enhances Wnt3a-dependent low density lipoprotein receptor related protein 6 phosphorylation, β-catenin protein accumulation and nuclear translocation in human embryonic kidney (HEK293) cells (29). Additionally, Fzd5 enhances the activity of SFRP1 stimulated Wnt3a in L cells while SFRP2 enhances Wnt3a activity in a dose-dependent manner in HEK293, C57MG and L cells (30). Retinoic acid extracted from vitamin A is essential for mammalian development, and vitamin A deficiency results in significantly reduced expression levels of SFRP1 in embryos (31). In addition, sequence and structure bioinformatics analysis tools are successful at predicting the potential inhibitors of SFRP4, including cycloformylhydrazide, clopramide and perindopril (16).

## Wnt Signaling Pathway

**Figure 2:**
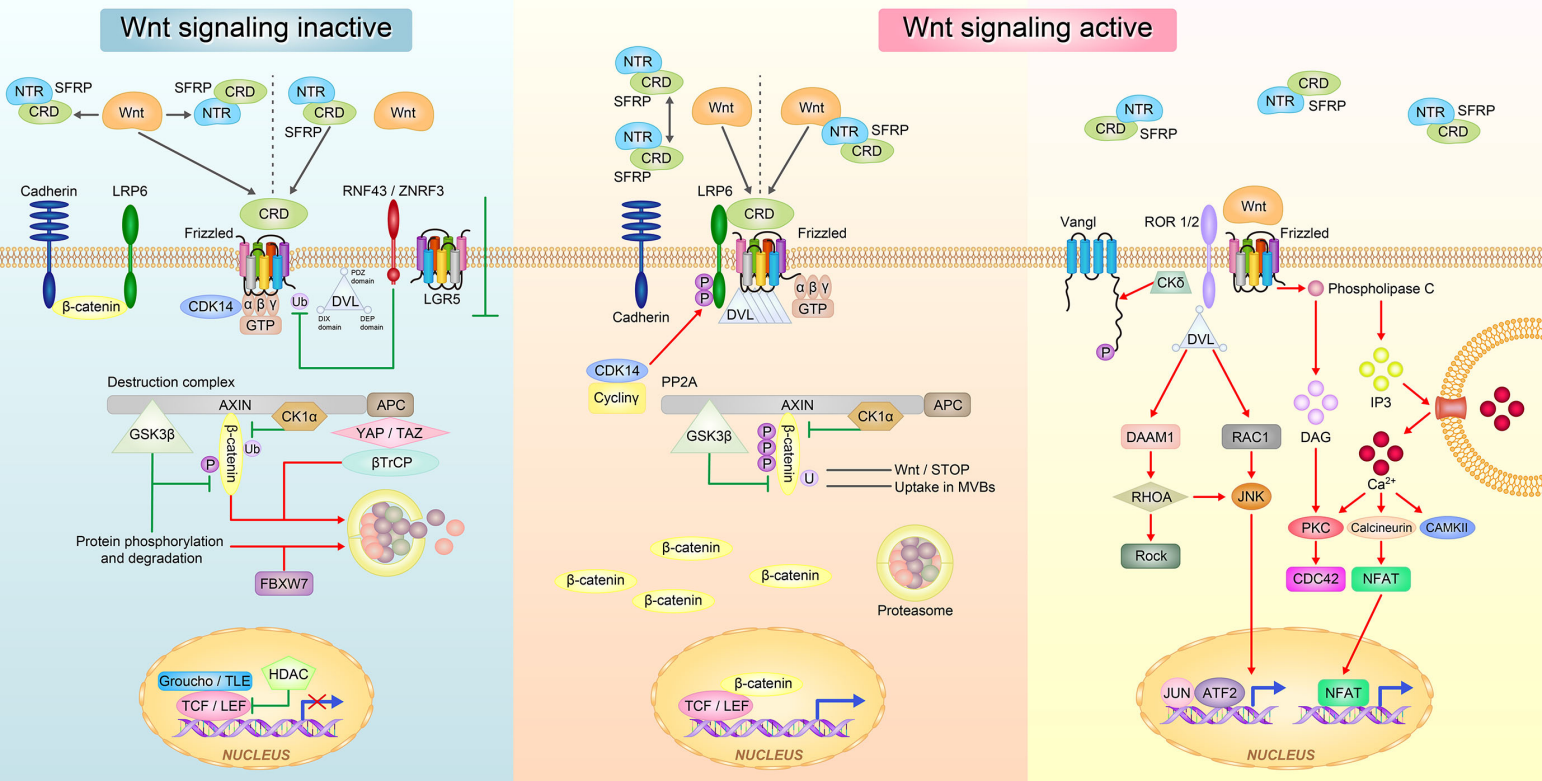
The Wnt signaling pathway regulated by SFRPs. In the inactive state of Wnt, SFRPs sequestered Wnt proteins *via* either CRD or NTR domain and prevent them from binding to the frizzled receptor. SFRPs can also bind to Frizzled receptor *via* CRD domain to block Wnt signal transduction (left). In the active state of Wnt, SFRPs form inactive complexes by their own and Wnt proteins directly bind to Frizzled receptor; or SFRPs form complex with Wnts and interact with Frizzled receptor to promote Wnt signal transduction (right). The Wnt proteins then activate at least three different downstream signaling pathways: the canonical Wnt-β-catenin, the non-canonical planar cell polarity (PCP) and the Wnt-Ca^2+^ pathways.

Wnt signaling has three typical pathways: canonical Wnt/β-catenin signaling pathway that controls cell fate determination and two non-canonical pathways that control cell movement and tissue polarity, namely, the Wnt/Ca^2+^ signaling pathway and planar cell polarity (PCP)/non-canonical c-Jun N-terminal kinase (JNK) signaling pathway (32, 33) (**Figure 2**).

In the canonical Wnt/β-catenin pathway, Wnts bind to the membrane receptor Fzd to inactivate the β-catenin degradation complex that consists of adenomatous polyposis coli/Axin/glycogen synthase kinase 3 beta (GSK3β) through disheveled (Dvl). During this process, β-catenin is not phosphorylated at specific serine and threonine residues, and it dissociates form the complex, binds to the T cell factor family of proteins, and translocates to the nucleus to transactivate target genes such c-myc and Cyclin-D1. In the absence of Wnt, β-catenin is phosphorylated by GSK3β or casein kinase and presented to the β-transduction repeat containing protein adenomatous polyposis coli/Axin proteins for ubiquitin mediated proteasomal degradation. For example, overexpression of SFRP4 promoted human adipose tissue derived mesenchymal stem cells differentiation and lipid accumulation by antagonistic inhibition of the Wnt/β-catenin signaling pathway activated by Wnt3a (34).

In the Wnt/Ca^2+^ signaling pathway, Wnts bind to Fzd to activate Dvl, which leads to an increase in intracellular Ca^2+^ and the activation of protein kinase C in response to distinct groups of Wnt ligands and Fzd receptors. Increased intracellular Ca^2+^ leads to a secondary activation of protein kinase C and calmodulin kinase II (35). For example, when identifying the role of SFRP4 in islet cells, we discovered that overexpression of SFRP4 activates the Ca^2+^ signaling pathway, enhances endocytosis, weakens exocytosis and decreases insulin secretion in islet cells (36).

The Wnt/PCP JNK signaling pathway uses Fzd and Dvl, but does not cause GSK-3β stabilization or calcium influx to regulate planar cell polarity (37). In vertebrates, Wnt/PCP signaling is thought to control polarized cell movements during gastrulation and neurulation (37). Wnt11 combined with fzd7 regulates projejunal motility in vertebrates, SFRP5 antagonizes the effect of Wnt5a in adipose tissue and activates the Wnt/PCP JNK signaling pathway by binding membrane receptors (38).

## Effect of SFRPs on Metabolic Disease

**Figure 3:**
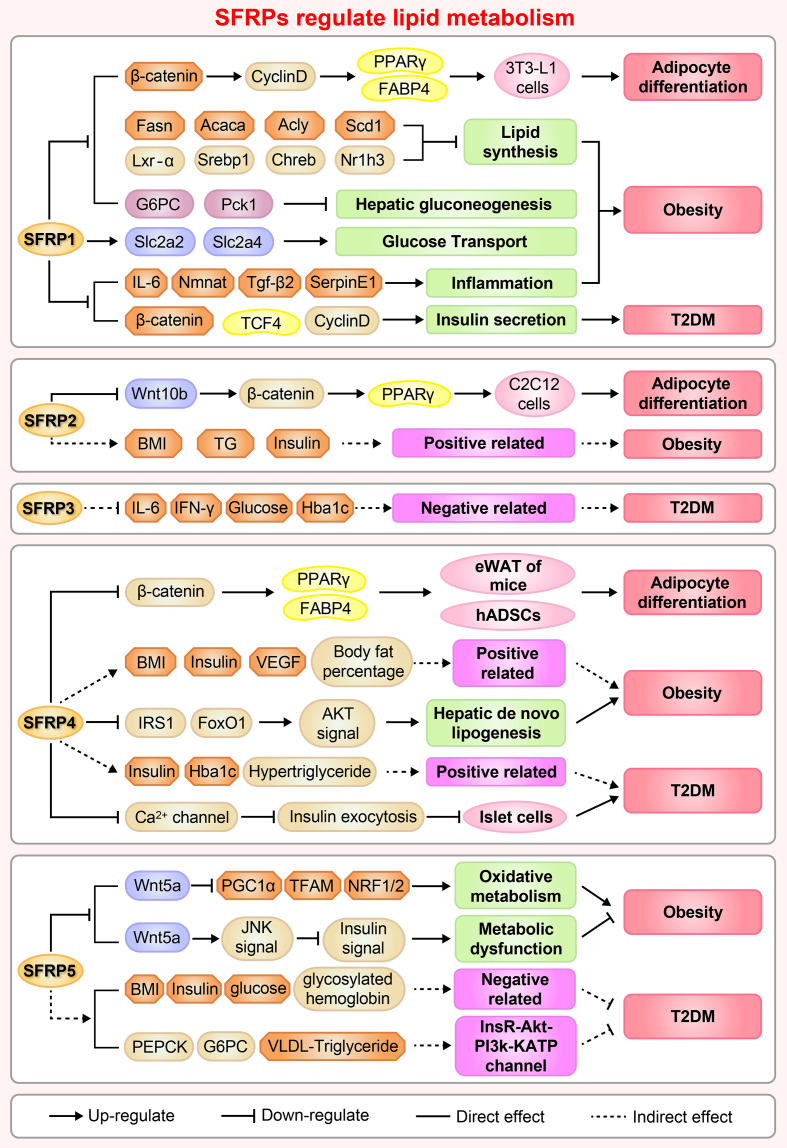
A brief overview of the molecular mechanisms by which SFRPs regulate lipid metabolism and cardiovascular disease. The figure shows the potential mechanisms by which how SFRPs modulate adipocyte differentiation, obesity and type 2 diabetes mellitus (T2DM). Acaca, acetyl-Coenzyme A carboxylase alpha; Acly, ATP citrate lyase; Akt, AKT serine/threonine kinase 1; Rho, rhodopsin; BMI, body mass index; Chreb, cAMP responsive element binding protein; FABP4, fatty acid binding protein 4; Fasn, fatty acid synthase; Foxo1, forkhead box O1; G6PC, glucose-6-phosphatase, catalytic; hADSCs, adipose mesenchymal stem cells; Hba1c, Glycosylated hemoglobin; IFN-γ, interferon-γ; IL-6, leukotriene-6; IRS1, insulin receptor substrate 1; Lxr-α, LexA regulated function-α; Nmnat, Nicotinamide mononucleotide adenylyltransferase; Nr1h3, nuclear receptor subfamily 1 group H member 3; NRF1/2, nuclear respiratory factor 1/2; Pck1, phosphoenolpyruvate carboxykinase 1, cytosolic; PCP, Pupal cuticle protein; PEPCK, phosphoenolpyruvate carboxykinase; PPARγ, peroxisome proliferator activated receptor-γ; BNP, natriuretic peptides A-like; Scd1, stearoyl-Coenzyme A desaturase 1; SerpinE1, serpin family E member 1; SFRP1, secreted frizzled related protein1; Slc2a2/4, solute carrier family 2 (facilitated glucose transporter), member 2/member4; Srebp1, sterol regulatory element binding protein 1; TCF4, transcription factor 4; TFAM, transcription factor A, mitochondrial; TG, triglyceride; Tgf-β2, transforming growth factor β 2; VEGF, vascular endothelial growth factor.

Adipocyte differentiation is closely related to glucose and lipid metabolism and regulates IR, T2DM and hyperlipoidemia (39). Adipogenesis is a tightly regulated cellular process involving multiple transcription factors and signaling pathways, including the Wnt signaling pathway and regulators of SFRPs (40). For example, SFRP1, SFRP2 and SFRP4 are adipokines, which related to insulin sensitivity and affect the secretion of interleukin-1, monocyte chemoattractant protein-1 and adiponectin (41). The regulation of adipocyte differentiation by SFRPs is shown in **Figure 3**.

### Adipocyte Differentiation

The functions of adipose tissue include lipid storage, energy balance and the sharing of insulin and other hormone signals. Adipogenesis is a process in which adipocytes develop from adipose tissue derived progenitor cells into adipose tissue (42). A series of studies have shown that SFRP1 contributes to adipogenesis in multiple cells. Overexpression of SFRP1 promotes 3T3-L1 cell differentiation in response to extracellular Wnt inhibitors (9). Conversely, SFRP1 deficiency inhibits adipogenesis and body fat in aged male mice (43). Moreover, SFRP1 mRNA expression dramatically increases in Grave’s ophthalmopathy caused by inflammation and an increased volume of the orbital adipose tissue (44). Meanwhile, inhibition of the Wnt/β-catenin singling pathway by Dac1 knockdown reversed SFRP1 induced impairment of adipogenesis (45), suggesting that SFRP1 is a negative regulator of adipose differentiation. The potential molecular mechanism involves GSK3 (an upstream regulator) activation of the signal transducer and activator of transcription 5 by phosphorylation, which then binds and regulates the SFRP1 promoter, coordinately modulates adipogenic regulator expression and antagonizes canonical Wnt signaling induced adipogenesis (46). These results provide insights on the molecular mechanism of adipogenesis and provide an evidence that different adipogenic regulators coordinately modulate adipocyte differentiation. Understanding the signaling pathways that mediate trans-differentiation between myoblasts and adipocytes is important to develop therapeutics for treatment of obesity and impaired metabolism (47). As a molecular switch between osteoblasts and adipogenesis in bone marrow, SFRP1 plays an important role as an autocrine and paracrine signaling molecule in mesenchymal stem cells (MSCs) or progenitor cells (48). For example, treatment of mouse bone marrow stromal cells with recombinant SFRP1 inhibits osteoblast differentiation of ST2 cells and promotes adipogenic differentiation in a dose-dependent manner (34, 49). Meanwhile, cyclic mechanical stretch inhibited myoblast trans-differentiation into adipocytes whereby SFRP2 treatment completely eliminated the inhibition of mechanical stretch on adipogenesis (50).

Our research indicates that SFRP4 expression during adipocyte differentiation is complex and varies among different tissues. Body size acquisition was contributed by depot-specific in adipose tissues and fat accumulation, as SFRP4 induces adipocyte maturation derived from visceral adipose tissue, meanwhile adipocytes from subcutaneous adipose tissue responds differently (51). Additionally, activation of Wnt signaling *via* knockdown of SFRP4 inhibits adipogenesis while the treatment with SFRP4 induces a 1.5-fold increase in lipid accumulation in human adipose derived mesenchymal stem cells (34, 52), these findings demonstrate an interaction between Wnt antagonism and Wnt activation during adipogenesis. As well known, Lysine demethylase 4a (KDM4A) is a novel epigenetic regulator of osteoblast and adipocyte differentiation that directly binds to the promoter of SFRP4, removes the histone methylation marker H3K9ME3, and reduces the DNA methylation of CpG in the promoter region of SFRP4. Concordantly, silencing of SFRP4 abolished the inhibition of KDM4A on osteogenic differentiation and the promotion of adipogenic differentiation induced by typical Wnt signal, these data have identified KDM4A as an upstream epigenetic regulator of SFRP4 in osteoblast and adipocyte differentiation (53).

In the early stage of 3T3-L1 preadipocytes, SFRP5 expression gradually increases at the start of preadipocyte differentiation. However, SFRP5 expression decreases significantly upon treatment with differentiation medium, indicating that SFRP5 expression is depend on the differentiation of adipocytes, maybe is a key regulator to improve insulin sensitivity by rosiglitazone or metformin in adipocyte (54). Remarkably, increasing doses of recombinant SFRP5, a competitive inhibitor of Wnt5a receptor, blocks the dedifferentiation of adipocytes when added to the co-culture medium (55). Subsequently, Zeng et al. revealed that SFRP5 promoter region (-2284 to -2263 bp) was under the transcriptional regulation of peroxisome proliferator-activated receptor gamma and is activated in 3T3-L1 adipocytes (56). Nevertheless, there is no significant difference in serum SFRP5 levels between obese and non-obese subjects, suggesting that SFRP5 exists only as a marker of mature adipocytes and does not regulate adipocyte differentiation (57).

### Obesity

SFRP1 increases in response to initial weight gain and decreases under conditions of extreme obesity in both humans and animals. Administration of high fat diet to SFRP1 knockout mice disturbs glucose homeostasis, induces inflammation and aberrantly elevates regulators of hepatic gluconeogenesis resulting in exacerbated weight gain, provide a new perspective that SFRP1 is a critical factor required for maintaining appropriate cellular signaling in response to the onset of obesity (43).

SFRP2 is highly expressed in omental adipose tissue compared to subcutaneous adipose tissue, and positively correlated with plasma insulin and body mass index (BMI). SFRP2 is expressed significantly in patients with impaired glucose tolerance compared to normal controls (34.2 ng/mL *vs.* 29.5 ng/mL) (58). However, diet induces significant changes in fat metabolism and SFRP2 expression in subcutaneous adipose tissue, indicating that weight loss regulates the expression of genes related to metabolic diseases in adipose tissue (59).

Previous study demonstrated that dietary macronutrients regulate insulin sensitivity and energy consumption, for example, the proportion of carbohydrate content is related to liver insulin sensitivity and does not affect serum SFRP4 level. This type of regulation suggests that dietary changes do not regulate the expression level of SFRP4 in serum (60). However, circulating SFRP4 in obese individuals is significantly higher than normal individuals, and is associated with body fat percentage, insulin sensitivity, vascular endothelial growth factor in abdominal subcutaneous adipose tissue, suggesting that abdominal subcutaneous adipose tissue maybe the main contributor of circulating SFRP4 (61). The increase of SFRP4 in visceral adipose tissue of obese men reduces the insulin receptor substrate 1 and fork head box O1 protein abundance in hepatocytes and inactivates protein kinase B (Akt) signaling pathway, resulting in fat breakdown in the liver and aggravated IR. Thus, SFRP4 may be an attractive target for alleviating the pathological development of type 2 diabetes and fatty liver (62).

SFRP5, an inhibitor of Wnt signaling, is tightly associated with obesity and weight gain in mice (63). SFRP5 is secreted by adipocytes and regulates the micro-environment of white adipose tissue under metabolic stress stimulation. First, Rulifson et al. reported that overexpression of SFRP5 increases fasting glucose and insulin levels and significantly impairs glucose intolerance in diet induced obese mice (64). Second, SFRP5 is significantly elevated in adipocytes during obesity, including db/db, ob/ob and high fat diet-fed mice. Meanwhile, SFRP5 deficiency resisted to diet induced obesity by stimulating mitochondrial oxidation activity and increasing SFRP1 compensation, which is mediated in part by peroxisome proliferator-activated receptor gamma coactivator 1 alpha and mitochondrial transcription factor A (65, 66). Third, obesity related complications also occurred in obese patients with low SFRP5 levels and pro-inflammatory infiltration of visceral tissue (67). Conversely, SFRP5 knockout mice did not exhibit a detectable phenotype when given normal food. However, administering a high calorie diet induces fat pad inflammation and systemic metabolic dysfunction, which is believed to be resulting from inhibition of JNK activation in macrophages and adipocytes *via* paracrine and autocrine mechanisms, respectively (38, 63). One important reason for this discrepancy may lie in differences between the construction sources of mice in contrast to mouse models in virous experiment. Furthermore, the phenotypes of obesity and diabetes in inbred mice with *SFRPs* homologous genes were significantly different (**Table 1**), for instance, there was no difference in SFRP5 expression levels in DBA/2J mice fed with high-fat diet, but almost 20 times higher in C57BL/6 mice, indicating that epigenetics regulate the phenotypes of obesity and fat swelling of inbred mice and this needs further investigation (107). In addition to studies using obesity animal model, the ratio of plasma Wnt5a/SFRP5 in obese patients is considered to be a more accurate evaluation index, which indicates that the decrease of SFRP5 concentration may be a direct factor leading to cardiovascular disease in obese patients (108). Fortunately, SFRP5 monoclonal antibody treatment significantly improved insulin sensitivity, restored islet β cell function and increased pro-insulin and C-peptide levels (64), which are consistent with those observed in the SFRP5^Q27stop^ mice described by Mori et al. (65).

**Table 1:** The overview of SFRPs knockout mice: Function and signaling pathways.

Genotype	Function	Signaling pathway	References
SFRP1^-/-^	Enhances trabecular bone formation in adults. Telencephalic Patterning, growth and differentiation. Promotes normal alveolar formation in lung development. Enhances mammary gland inflammation in response to obesity Mediates mammary epithelial apoptotic response to DNA damage. Maintains proper mammary gland development. Improves fracture healing Regulates the tumors progression in murine mammary epithelial cells. Regulates the progression of renal fibrosis. Regulates cycling activity and maintenance of hematopoietic stem cells. Leads to deterioration of cardiac function in aged mice. Regulates the cancer stem cells proliferation and metastasis in skin cancer. Contributes to the development of brain, kidney and skeleton.	Wnt signaling in osteoblasts Wnt and Notch signaling in hippocampal. Wnt and ERK signaling in lung tissue. Wnt and apoptosis in breast cancer of obesity. P53 target genes pathway in mammary epithelial cells. Wnt and downstream regulators in mammary gland. Wnt signaling in bone formation. Wnt and TGFβ signaling pathway. Non-canonical Wnt/PCP pathway. Extrinsic regulation of β-catenin. Wnt signaling pathway in cardiac tissue. EMT regulators and growth factor signaling. Wnt signaling pathway.	(68–80)
SFRP2^-/-^	Contributes to the development of primary aldosteronism. Controls fibrosis in myocardial infarction. Plays a critical role in proper distal limb formation. Modulates cell fate of the murine intestinal epithelium	Wnt signaling pathway. Enhanced procollagen C-proteinase activity. Wnt/β-catenin signaling pathway. Wnt/β-catenin signaling pathway	(81–84)
SFRP3^-/-^	Controls post-natal mammary gland morphogenesis. modulates the progressive weakness and muscle degeneration. As a molecular target of antidepressant treatments in rodent models. Regulates cell development of articular cartilage - subchondral bone unit. Contributes to the development of osteoarthritis. Reduces voluntary running exercise performance in mice. Regulates quiescent adult hippocampal neural stem cell activation. Promotes the osteogenesis	Wnt/β-catenin signaling pathway. Wnt/β-catenin signaling pathway. Wnt/β-catenin signaling pathway. Wnt/β-catenin signaling pathway. Increase the activity of MMPs. Increased Wnt activity and MMPs. Increased RGL activation in the dentate gyrus Reduced canonical but increased non-canonical Wnt signaling	(85–92)
SFRP4^-/-^	Results in high total area and greater trabecularization. Does not lead to altered serum or urine phosphate levels. Alters body size, food intake and energy expenditure in obese mice. Increases trabecular bone formation and unusually thin cortical bone in mice. Decreases periosteal and endosteal bone formation in mice.	None. None. None. Wnt and BMP signaling. Repression of the Ror2/Jnk cascade in osteoclasts.	(25, 93–96)
SFRP5^-/-^	Not essential for axis formation or foregut morphogenesis in the mouse. Ameliorates mouse liver fibrosis. Regulates early lympho-hematopoiesis in the maternal bone marrow. diminishes cardiac inflammation and protects the heart from ischemia-reperfusion injury	None. Inhibition of Wnt5a/Fz2 signaling. Strongly block B-lymphopoiesis. Block the JNK activation by induction of Wnt5a in bone marrow derived macrophage	(97–100)
SFRP1^-/-^/SFRP2^-/-^	Regulates anteroposterior axis elongation and somite segmentation during mouse embryogenesis Requires for normal male sexual development. Regulates retinal neurogenesis. Requires for maintenance in lens epithelial cells	Alter oscillations of Notch signaling. Disrupted non-canonical Wnt signaling. Act as negative modulators of ADAM10. Wnt/β-catenin signaling.	(101–104)
SFRP2^-/-^/SFRP4^-/-^	Negatively regulates ovarian follicle development.	Wnt signaling pathway in ovarian follicle development.	(105)
SFRP1^-/-^/SFRP2^-/-^ /SFRP5^-/-^	Regulates early Trunk Formation in Mouse.	Canonical and non-canonical pathways	(106)

ADAM10, a disintegrin and metalloproteinase 10; BMP, bone morphogenetic protein; DNA, deoxyribonucleic acid; EMT, epithelial to mesenchymal transition; ERK, extracellular signal-regulated kinase; Fz, frizzled; JNK, c-Jun N-terminal kinase; MMP, matrix metalloprotease; RGL, radial glia-like neural stem cell; Ror2, receptor tyrosine kinase like orphan receptor 2; SFRP, secreted frizzled-related protein; TGFβ, transforming growth factor beta; Wnt, wingless/integrated; Wnt/PCP, Wnt/planar cell polarity.

### Diabetes and IR

Adipose tissue, as an endocrine organ, secretes more than 300 cytokines to regulate the response of target cells including islet β cells (109). It has been reported that overexpression of SFRP1 inhibited the insulin secretion from islet cells which are co-cultured with human adipocytes (110). Otherwise, muscle SFRP3, acting as a new type of insulin sensitizer, negatively correlated with circulating inflammatory factors and positively correlated with insulin sensitivity, is expressed at low levels in skeletal muscles and serum of pre-diabetes and T2DM patients compared to healthy subjects (111).

Multiple clinical studies proved that SFRP4 is involved in the occurrence and development of T2DM, as elevated serum SFRP4 puts patients at three-folds higher risk of developing diabetes (10, 112–115). Serum SFRP4 concentration is independently and positively correlated with insulin, glycosylated hemoglobin, fasting triglyceride and hypertriglyceridemia levels (10, 113–115). Moreover, circulating SFRP4 and renin are significantly higher in gestational diabetes mellitus patients than healthy subjects. In addition SFRP4 and renin levels are positively correlated (116). Compared with the healthy individuals, patients with polycystic ovary syndrome showed higher levels of circulating SFRP4 which is positively correlated with IR, androgen, ovarian follicle number or ovarian volume (117). Multiples studies aimed to reveal the underlying mechanism of SFRP4 function. For instance, Mahdi et al. noted that interleukin-1β stimulated SFRP4 secretion impairs glucose tolerance and insulin secretion in islet cells of T2DM patients (36). However, Mastaitis et al. demonstrated that SFRP4 doesn’t regulate glucose homeostasis and β-cell quality in mice, as SFRP4 knockout mice exhibited the same level of impaired glucose tolerance, and the reason was tied to 5-fold compensation by islet β cells (25). microRNAs regulate adipose tissue functions, such as lipolysis, glucose and glycerol conversion and insulin sensitivity (118). The levels of miR-24, miR-30d and miR-146a are higher in abdominal adipose tissue of obese and T2DM patients compared to normal subjects, which positively correlates with the expression of SFRP4 (119). Another group discovered that miR-103a and miR-103b negatively regulate the expression of SFRP4 by regulating the 3’-noncoding region, suggesting that their mutual changes provide high sensitivity and specificity for the identification of patients with pre-diabetes (120).

In a series of clinical cases the analysis of circulating SFRP5 levels in T2DM patients lead to an opposite conclusion. Data showed that circulating SFRP5 level is significantly lower in obese, diabetic and adult latent autoimmune diabetes patients compared to normal subjects, and is negatively correlated with BMI, waist circumference, fasting blood glucose, glycosylated hemoglobin, insulin level and IR index, supporting the role of SFRP5 as a protective factor in the pathogenesis of diabetes (121–126). To the contrary, Canville et al. reported that the plasma level of SFRP5 was higher in newly diagnosed T2DM patients compared to pre-diabetic or normal subjects, suggesting SFRP5 is an independent risk factor for T2DM (127). The controversial results could be attributed to the following reasons: firstly, medications used in managing diabetes may affect SFRP5 secretion, as Lu et al. reported a clinical analysis including two medications (rosiglitazone and metformin) (122); secondly, the conclusion is based on a small number of cases and the distribution was not equal in T2DM patients and normal control (126); thirdly, age, gender and BMI may affect SFRP5 levels, which were excluded in Canville’s study. Central SFRP5 also plays an important role in liver lipid metabolism mainly in reducing food intake, increasing energy consumption, and inhibiting liver glucose flow and very low-density lipoprotein-triglyceride production. The mechanism underlying these processes is likely that SFRP5 dependent neural circuit participates in hypothalamic insulin signaling pathway, and inhibits N-methyl-D-aspartate receptor or dorsal vagal complex ATP-sensitive potassium channel mediated hepatic vagus nerve conduction, which then reduces the activities of liver adipogenesis related enzymes and inhibits the secretion of hepatic glucose and very low-density lipoprotein-triglyceride (128). *In vitro*, glucose inhibits phosphatidylinositol 3-kinase/Akt pathway and reduces the expression of SFRP5 to slow the proliferation of rat islet β cells, indicate that SFRP5 may serve as a target to expand functional pancreatic islets in diabetic patients (129).

## The Effect of SFRPs on Cardiovascular Disease

**Figure 4:**
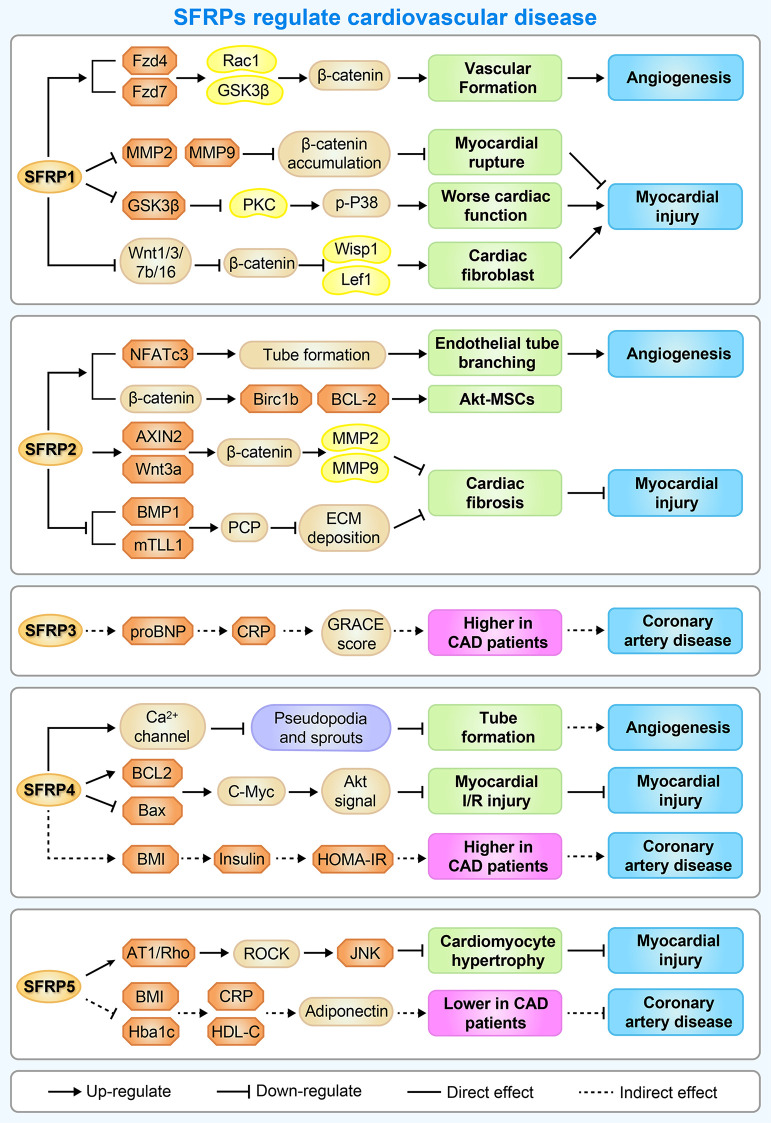
A brief overview of the molecular mechanisms by which SFRPs regulate cardiovascular disease. The figure shows the potential mechanisms by which how SFRPs modulate cardiovascular diseases including angiogenesis, myocardial injury and coronary artery disease. AT1, arginine ADP-ribosyltransferase 1; AXIN2, axin protein 2; BAX, BCL2 associated X, apoptosis regulator; BCL-2, B cell leukemia/lymphoma 2; Birc1b, NLR family, apoptosis inhibitory protein 2; BMP1, bone morphogenetic protein 1; C-Myc, MYC proto-oncogene, bHLH transcription factor; CRP, C-reactive protein; ECM deposition, Subclass B3 metallo beta lactamase gene family; Fzd4/7, frizzled class receptor 4/7; HDL-C, High-density lipoprotein; HOMA-IR, Homeostasis model assessment-IR; JNK, c-Jun NH2-terminal kinase; Lef1, lymphoid enhancer binding factor 1; MMP2, matrix metalloproteinase 2; NFATc3, nuclear factor of activated T cells, cytoplasmic, calcineurin dependent 3; PKC, protein kinase C; Rac1, Rac family small GTPase 1; ROCK, Rho-associated coiled-coil containing protein kinase; WISP1, WNT1 inducible signaling pathway protein 1; Wnt1, wingless-type MMTV integration site family, member 1.

Cardiovascular disease remains a leading cause of mortality worldwide. It is widely accepted that unbalanced cardiovascular remodeling and ischemic injury are the major pathological processes involved in cardiovascular diseases, leading to adverse prognoses. There is therefore a great need for a novel approach to prevent cardiovascular remodeling and ischemic injury. Presently, multiple studies suggest that SFRPs may represent novel therapeutic targets (**Figure 4**).

### Angiogenesis

Dufourcq et al. demonstrated that SFRP1 inhibits Wnt signaling to increase the angiogenesis of mesenchymal cells, glioma cells and chorioallantoic membrane, making the blood vessels larger, longer and more mature and promoting the migration of endothelial cells (ECs) and the formation of catheters (130). Alternatively, overexpression of SFRP1 inhibits the proliferation of ECs and smooth muscle cells by delaying the G1 to S phase transition, altering the Wnt frizzled pathway and controlling the proliferation and angiogenesis of muscle after ischemia (39). For these reasons, SFRP1 would be of interest for cardiac surgeons using angiogenic therapy in ischemic heart diseases in non-revascularizable patients. Additionally, SFRP1 interacts with Wnt receptors Fzd4 and Fzd7 to promote ECs’ cytoskeletal reorganization and increases neovascularization in ischemia-induced angiogenesis in mouse hindlimbs (131). It is worth mentioning that nuclear factor of activated T cells 3 is required for SFRP2 induced endothelial tube formation (132).

Recent studies indicate that SFRP4 inhibits the formation of pseudopodia and sprouts as well as disrupts the stability of ECs ring *via* antagonizing the Wnt signaling pathway and inhibiting the nuclear translocation of β-catenin. This inhibition drives ECs toward apoptosis and inhibits angiogenesis by increasing cellular levels of reactive oxygen species (133). The results of SFRP4 domain analysis revealed that CRD inhibits the tube formation by ECs, NLD could promote EC death, inhibits angiogenesis and increases intracellular calcium levels in ECs by activating non-classical Ca^2+^ Wnt signaling pathway in SFRP4 mediated angiogenesis inhibition, which is suggestive of alternative antiangiogenic downstream targets of canonical Wnt signaling (134).

### Myocardial Injury

SFRP1 regulates myocardial differentiation and the formation of large and small myocardium during embryonic development by inhibiting Wnt6 signaling pathway (135). Besides, SFRP1 protects rat cardiomyoblasts from hypoxia/re-oxygenation injury or transverse aortic constriction induced heart failure by blocking the Wnt signaling pathway (136, 137). In addition, overexpression of SFRP1 reduces myocardial rupture, early leukocyte infiltration and apoptosis index as well as increases collagen deposition and capillary density, preferentially improved the myocardial function and infarct size after myocardial infarction (138). In brief, SFRP1 is not only controls the size of the differentiating heart muscle primarily by regulating cell fate within the cardiac mesoderm between muscular and non-muscular cell lineage, but also through direct or indirect interaction with different phases of infarct healing, reduced infarct size and improved cardiac function. Contrary to previous study, during ischemic preconditioning, SFRP1 overexpression reduces GSK-3β phosphorylation levels in the heart. Compared with the littermates, the SFRP1 transgenic mice show larger infarct size and worse cardiac function (139). Conversely, SFRP1 deficiency results in abnormal cardiac structure, dysfunction, ventricular dilatation and hypertrophy, deterioration of cardiac function and a large number of myocardial fibrosis, accompanied with an increase in expression of Wnt ligands (Wnt1, 3, 7b, and 16) and Wnt target genes (Wisp1 and Lef1) in the heart of aged mice (78).

The protective effect of SFRP2 on the heart can be divided into two aspects: one is to promote the proliferation and differentiation of cardiac progenitor cells, the other is functional diversity and signal complexity of SFRP2 in cardiac fibrosis. Typically, overexpression of SFRP2 increases the proliferation rate, reduces apoptosis, inhibits the combination of osteoblasts and chondrocytes and promotes self-renewal ability of MSCs by inhibiting Wnt and bone morphogenetic protein (BMP) signaling pathways to achieve better tissue transplantation and wound healing (140). The ability to regenerate and replace dead myocardium after heart injury is limited, and overexpression of Akt by bone marrow MSCs significantly reduces the infarct size of myocardial injury and restores myocardial function in rodents (141). Overexpression of AKT in MSCs up-regulates SFRP2 by 100 fold to promote myocardial survival and repair after ischemic injury (142). Bone marrow MSCs transplantation is an ideal method to treat ischemic injury, however the insufficient survival rate of transplanted cells in host tissues is a main obstacle halting the progress of cell therapy. Injection of SFRP2-MSCs into the myocardium around myocardial infarction enhances the transplantation, increases the vascular density, decreases the infarct area and enhances the cardiac function after myocardial injury in mice (143). Cardiac progenitor cells are essential for replacement of lost mature cells during injury or turnover (144). Firstly, SFRP2 binds to Wnt6 and inhibits the classic Wnt pathway, slows the proliferation of cardiac progenitor cells and activates Wnt/JNK signaling pathway to induce the differentiation of cardiac progenitor cells (145). Secondly, SFRP2 reduces Wnt3a transcription to prevent mesoderm formation and cardiomyocyte differentiation (146). Collectively, the researchers demonstrate the novel function of SFRP4 in regulating the dynamic process of cardiac progenitor cells proliferation and differentiation, as well as providing new insights into the mechanisms of Wnt signaling in cardiac differentiation. SFRP2 upregulates the expression of Axin2 and Wnt3a to promote the growth of fibroblasts, and increases the expression of matrix metalloproteinase to reduce chronic fibrosis and heart failure after myocardial infarction (147). Antibody blocking SFRP2 activates Axin2 to improve myocardial function and inhibits myocardial fibrosis, which may be a specific target of anti-fibrosis therapy (148). The underlying molecular mechanism by which SFRP2 promotes cardiac fibro-calcification is probably through the coordinate activation of tolloid-like metalloproteinases and TNAP (149). Correspondingly, knockdown of SFRP2 markedly reduces fibrosis in the infracted heart (82). In details, SFRP2 enhances the activity of BMP-1 at low concentrations (10-20 nm), while at high concentration (100 nm) it specifically inhibits PCP activity, BMP activity and collagen maturation *in vitro*. In addition, SFRP2 inhibits the maturation of procollagen and increases the accumulation of soluble type I procollagen in a dose-dependent manner, but has no effect on collagen synthesis in total cell lysates (150). In conclusion, SFRP2 inhibits myocardial injury induced fibrosis in a dose-dependent manner.

Schumann et al. demonstrated that SFRP3 and SFRP4 are highly expressed in human left ventricle during heart failure, they also enhance the apoptosis susceptibility by antagonizing Wnt signal transduction (151). Moreover, patients with heart failure showed increased levels of circulating SFRP3 which associated with a poor outcome (152). Furthermore, intermediate serum levels of SFRP3 increases survival rate in elderly patients with ischemic chronic heart failure, indicating that balanced Wnt activity may play a protective role in clinical heart failure patients (153). As well known, both transient and permanent ischemic injuries lead to cell death, scar formation and tissue remodeling. Upregulation of SFRP4 in ischemic heart improves cardiac function after permanent and transient ischemic injuries through reduction of cellular scar formation (154). Conversely, SFRP4 knockdown decreases lactate dehydrogenase and creatine kinase levels, increases ventricular function by activating the Akt signaling pathway in the myocardium to alleviate ischemia-reperfusion injury by reduce protein expression of Bax, active caspase 3, and increase Bcl-2 and c-Myc in cardiac tissue (155). Altogether, administration of SFRP4 interferes with canonical Wnt signaling that could mediate the formation of acellular scar and consequently contributes to the prevention of aggravation of cardiac function.

The infarct size of SFRP5 knockout mice is significantly larger than wild-type mice. In addition, Wnt5a positive macrophages and inflammatory cytokines increases significantly after ischemia-reperfusion in SFRP5 knockout mice compared to the wild type mice (100). Serum SFRP5 is significantly higher in patients with acute ST segment elevation myocardial infarction (STEMI) compared to patients without CHD, and is negatively correlated with the levels of high-sensitivity cardiac troponin I and C-reactive protein (156). Consequently, angiotensin II increases the expression of SFRP5 in a time and dose-dependent manner, as SFRP5 inhibits the expression of natriuretic peptide b and tumor necrosis factor alpha in hypertrophic cardiomyocytes through AT1 receptor/Rho/Rock1/JNK signaling pathway, which plays an important role in the pathological process of cardiac hypertrophy (157). Otherwise, the ancestral heart is accompanied by the generation and remake of progenitor cells after the acquisition of pulmonary circulation. The expression of SFRP5 begins at the lateral side of the crescent and continues to be expressed at the venous pole, indicating that the outflow tract, left ventricle, atrium and venous sinus originates from the common progenitor cells, while the right ventricle is not (158).

### Coronary Artery Disease

Wnt signaling is involved in atherosclerotic plaque formation directly and indirectly by modulating cardiovascular risk factors. Baseline systemic SFRP1 levels are significantly higher in patients with cardiovascular events compared to healthy controls (159). SFRP3 is a predictor of all-cause mortality and re-hospitalization due to stroke in a large population of acute coronary syndrome patients with long-term follow-up. The association between adverse events and intermediate SFRP3 levels is further supported when combining intermediate SFRP3 levels and advanced GRACE score, further supports a role for the Wnt pathways in the progression of clinical atherosclerosis involving the regulation of soluble Wnt modulators (160).

SFRP4 mRNA and protein expression are significantly higher in CAD patients compared to non-CAD in epicardial adipose tissue (EAT) and plasma. Moreover, EAT SFRP4 mRNA levels and plasma SFRP4 concentrations are independently associated with the presence of CAD by multivariate linear regression analysis (161). Additionally, the levels of SFRP4 in control group are significantly lower than those in T2DM, CHD and T2D+CHD, indicating that SFRP4 may be a predictive marker for atherosclerosis particularly in diabetic patients (162).

Additional report indicates that the serum level of SFRP5 is significantly lower in patients with CAD compared to patients without CAD, and is negatively correlated with the severity of CAD (163). In order to further clarify the relationship between SFRP5 and cardiovascular disease risk factors, a large population-based cohort study was conducted and demonstrated that the increase of serum SFRP5 is negatively correlated with various risk factors of T2DM and cardiovascular disease, indicating that SFRP5, as a new biomarker, needs further investigation as a mediator in the prevention of cardiac metabolic diseases (24, 164). The ratio of SFRP5 to Wnt5a is lower in EAT and serum in CAD patients compared to healthy subjects. Besides, the serum level of SFRP5 is also lower in CAD patients compared to the healthy subjects and negatively associates with the presence of CAD. SFRP5 is secreted by visceral fat and its local concentration in EAT may greatly exceed that in subcutaneous adipose, indicating that low SFRP5 and high Wnt5a levels are associated with the presence of CAD, independent of other conventional risk factors (165). Adiposity, the level of c-reactive protein, leptin and C-C motif chemokine ligand 2 are higher in Mexican Americans consuming a diet high in sugar sweetened beverages, however, the expression of SFRP5 is lower in participants with diet rich in fruits, vegetables and includes low sugar sweetened beverages intake, this reveals that diet contributes to adiposity and pro-inflammation (166).

## Conclusion and Perspective

Over the past 30 years, SFRPs family members have become a popular research topic. The expanding family of SFRPs, with their multiple functions, provides abundant research targets and contributes to the development of novel therapies. In this review, we summarize the influence of SFRPs protein on physiological and pathological processes related to lipid metabolism and cardiovascular protection (**Figures 3**, **4**). Although many important scientific problems have been solved since SFRPs proteins were first described, there are still many problems to be solved. (1) In the study of adipogenesis in mice, what makes SFRPs specific in regulating adipogenesis and what is the mechanism leading to this difference? (2) In clinical research, the physiological levels of SFRPs proteins changed greatly (**Tables 2**, **3**). It is necessary to further clarify whether the SFRPs levels are regulated during the development of obesity and diabetes mellitus since there is still controversy on whether the SFRPs levels are higher or lower than a specific threshold to affect metabolism. (3) In the development of cardiovascular disease, the expression levels of SFRPs protein are changed. In the future, whether SFRPs can be used as markers for the diagnosis or prediction of cardiovascular diseases, such as CHD, atherosclerosis, *etc*. (4) Identifying SFRPs receptors is still a major challenge and there is a need to provide in-depth insights into the signaling pathways controlled by each of the SFRPs and the unique biological functions they mediate.

**Table 2:** The clinic study about the concentration of SFRPs in patients with metabolism disease.

Genotype	Study population (number)	Concentration of SFRPs	References
SFRP4	Obese Patients Study	Obese: 137.8±33.6 ng/mL; Lean: 64.1±23.8 ng/mL.	(61)
	Lean (n=8); obese (n=12).		
SFRP4	Obese Patients Study	PW: 1086±167 pg/mL; LF: 1307±181pg/mL; LGI: 1346 ±211 pg/mL; VLC: 1220 ±205 pg/mL.	(60)
	LF (n=21); LGI (n=21); VLC (n=21).		
SFRP4	Diabetic Study	NGT: 95.46 ± 20.13 ng/mL; IGT: 141.64 ± 40.46 ng/mL; T2DM: 184.38 ± 61.34 ng/mL.	(113)
	NGT (n=42); IGT(n=52); T2DM (n =56).		
SFRP4	Diabetic Study	Control: 8.8 ± 3.0 ng/mL; CO-T1D: 16.9 ± 4.5 ng/mL; T2D: 37.1 ± 26.7 ng/mL; LADA: 15.6 ± 6.2 ng/mL; T1D: 24.6 ± 17.9 ng/mL.	(114)
	Control (n=30); T1D (n=46); T2D (n=55); CO-T1D (n=30); LADA (n=37).		
SFRP4	Diabetic Study	NGT: 0.170 (0.12–0.45) ng/mL; IGT: 0.183 (0.13–0.70) ng/mL; T2DM: 0.282 (0.11-2.51) ng/mL.	(115)
	NGT (n=36); IGT (n=34); T2DM (n=82).		
SFRP4	Pregnant with Diabetic Study	Control: 5.59 ± 3.32 ng/mL; GDM: 4.05 ± 2.15 ng/mL.	(116)
	Control (n=41); GDM (n=35).		
SFRP4	Women with PCOS	Control: 5.87 ± 1.91 ng/mL; PCOS: 3.72 ± 1.29 ng/mL.	(117)
	Control (n=80); PCOS (n=80).		
SFRP5	Diabetic Study	NGT-NW :13.12±3.62 ng/mL; NGT-OB: 9.46±2.70 ng/mL; T2DM-NW: 10.12±3.45 ng/mL; T2DM-OB: 6.70±2.34 ng/mL; Men: 9.71±3.86 ng/mL; Women: 10.01±3.82 ng/mL.	(121)
	NGT-NW (n=46); NGT-OB (n=43); T2DM-NW (n=42); T2DM-OB (n=45).		
SFRP5	Diabetic Study	T2DM: 9.4±9.0 ng/mL; non-diabetics: 7.4±10.9 ng/mL; Male: 9.8±10.7 ng/mL; Female:7.3±8.2 ng/mL.	(122)
	T2DM (n=82), non-diabetics (n=42).		
SFRP5	Diabetic Study	Controls: 22.98±12.36 ng/mL; T2DM: 14.14±11.91 ng/mL; LADA: 14.82±11.27 ng/mL.	(124)
	Controls (n=40); T2DM (n=58); LADA (n=22).		
SFPR5	MetS Study	Control subjects: 61.6±23.2 µg/L; MetS patients: 49.1±17.2 µg/L.	(125)
	Control (n=194); MetS patients (n=90).		
SFRP5	Diabetic Study	Controls: 10.4 (6.7-16.6) ng/mL; T2DM: 15.6 (9-24.5) ng/mL; prediabetic subjects: 9.8(5-14.2) ng/mL.	(127)
	Controls (n=70); T2DM (n=70); prediabetic subjects (n=70).		

CO-T1D, controls, age matched for patients with type 1 diabetes; GDM, gestational diabetes mellitus; IGT, impaired glucose tolerance; LADA, latent autoimmune diabetes of the adult; LF, low fat; LGI, low glycemic index; MetS, metabolic syndrome; NGT, normal glucose tolerance; NGT-NW, normal glucose tolerance-Normal weight; NGT-OB, normal glucose tolerance-obese; PCOS, polycystic ovary syndrome; PW, pre-weight; T1D, type 1 diabetes; T2, type 2 diabetes; T2DM, type 2 diabetes mellitus; T2DM-NW, type 2 diabetes mellitus-normal weight; T2DM-OB, type 2 diabetes mellitus-obese; VLC, very low carbohydrate.

**Table 3:** The clinic study about the concentration of SFRPs in patients with cardiovascular disease.

Genotype	Study population	Concentration of SFRPs	References
SFRP1	Cardiovascular disease: Controls (n=157); Patients with cardiovascular events (n=).	Control: 2609.0 [1926.1-3234.9] pg/mL; CVD: 3221.8 [2352.8-3811.0] pg/mL.	(159)
SFRP3	HF study: Controls (n=25); HF patients (n= 153).	Control:1.83 (1.40, 2.46) ng/mL; HF patients: 2.83 (2.46, 3.62) ng/mL.	(167)
SFRP4	Cardiovascular disease: CAD patients (n=504).	Control: 12.21 (3.64 – 41.2) μg/L; CAD patients: 11.21 (9.17, 13.86) μg/L.	(10)
SFRP4	Cardiovascular disease: Non-CAD(n=30); CAD (n=30).	non-CAD: 14.5 ± 2.3 ng/mL. CAD: 16.8 ± 3.3 ng/mL;	(161)
SFRP4	Cardiovascular disease: Control (n=35); DM (n=37); CAD (n=34); CAD+DM (n=36).	Control:1.41 (1.24–1.67) ng/mL; DM: 1.70 (1.51–2.45) ng/mL; CAD: 1.58 (1.31–2.55) ng/mL; CAD+DM: 2.02 (1.47–3.47) ng/mL.	(162)
SFRP5	Cardiovascular disease: non-CAD (n=57); CAD (n=128).	non-CAD: 52.4 [29.6] ng/mL; CAD: 47.7 [26.6] ng/mL;	(163)
SFRP5	Cardiovascular disease: non-CAD (n=29); CAD (n=58).	non-CAD: 21.27±3.38 ng/mL; CAD: 16.23±3.17 ng/mL.	(165)
SFRP5	MI study: no-CAD (n=35); STEMI (n=85).	no-CAD: 19.8 ng/mL; STEMI: 23.3 ng/mL.	(156)

CAD, Cardiovascular disease; DM, Diabetes mellitus; HF, Heart failure; MI, Myocardial infarction; SFRP, Secreted frizzled-related protein; STEMI, ST-Elevation Myocardial Infarction.

We conclude that our current knowledge about the role of SFRPs family proteins in IR, β cell dysfunction, type 2 diabetes and the natural cause of cardiovascular disease is still limited. The same is true of their potential relevance in regulating disease progression to improve metabolic process. However, we look forward to more basic and clinical research on SFRPs in the future, which can be used as a bedding for clinical treatment. Although previous studies have shown that SFRPs family proteins have the potential to be used as biomarkers, so far, the relevant research conclusions are not enough to support their use as molecular markers to predict diseases. Due to the contradictory conclusions of several similar studies (25, 36, 38, 65), more basic and clinical studies are still needed in the future, and more theoretical basis is needed to prove its feasibility as a molecular marker.

## Author Contributions

HG, LX and XG conceived this manuscript. HG, JZ and ZH collected and prepared the related references. HG, JZ and JL drafted the manuscript. HG, HX and ZH drew the figures. HX and QY performed data analysis and tabulation. LX, and XG and QY supervised and revised the manuscript. HG and ZH summarized the contents of the article and drew the graphical abstracts. All authors contributed to the article and approved the submitted version.

## Funding

This work was supported by grants from National Natural Science Foundation of China (grant: 81900399), Natural Science Foundation Project of Shaanxi Province (grants: 2020JZ-56, 2021JQ-785, 19JS060, 2016JM8122, 2020JM-605) and State Administration of Traditional Chinese Medicine of Shaanxi Province (grant: 2019-ZZ-JC034), the Key Research and Development Plan of Shaanxi Province (grant: 2018SF-266), Xi’an Medical University Scientific Research Fund (grant: 2018DOC12, 2016RCYJ01).

## Conflict of Interest

The authors declare that the research was conducted in the absence of any commercial or financial relationships that could be construed as a potential conflict of interest.

## Publisher’s Note

All claims expressed in this article are solely those of the authors and do not necessarily represent those of their affiliated organizations, or those of the publisher, the editors and the reviewers. Any product that may be evaluated in this article, or claim that may be made by its manufacturer, is not guaranteed or endorsed by the publisher.

## Abbreviations

Akt, protein kinase B; BMP, bone morphogenetic protein; BMI, body mass index; CHD, coronary heart disease; CRD, cysteine rich domain; Dvl , disheveled; EAT, epicardial adipose tissue; ECs, endothelial cells; Fzd, frizzled; GSK3β, glycogen synthase kinase 3 beta; HEK293, human embryonic kidney; IR, insulin resistance; JNK, c-Jun N-terminal Kinase; KDM4A, Lysine demethylase 4a; MetS, Metabolic syndrome; MSCs, mesenchymal stem cells; NTR, netrin-like domain; PCP, pathway and planar cell polarity; SFRP, secreted frizzled related protein; STEMI, ST segment elevation myocardial infarction; T2DM, type 2 diabetes mellitus.
